# Folding Rate Optimization Promotes Frustrated Interactions in Entangled Protein Structures

**DOI:** 10.3390/ijms21010213

**Published:** 2019-12-27

**Authors:** Federico Norbiato, Flavio Seno, Antonio Trovato, Marco Baiesi

**Affiliations:** 1 Department of Physics and Astronomy, University of Padova, Via Marzolo 8, I-35131 Padova, Italy; federico.norbiato@gmail.com (F.N.); flavio.seno@pd.infn.it (F.S.); trovato@pd.infn.it (A.T.); 2 INFN, Sezione di Padova, Via Marzolo 8, I-35131 Padova, Italy

**Keywords:** protein folding, entanglement, topological frustration

## Abstract

Many native structures of proteins accomodate complex topological motifs such as knots, lassos, and other geometrical entanglements. How proteins can fold quickly even in the presence of such topological obstacles is a debated question in structural biology. Recently, the hypothesis that energetic frustration might be a mechanism to avoid topological frustration has been put forward based on the empirical observation that loops involved in entanglements are stabilized by weak interactions between amino-acids at their extrema. To verify this idea, we use a toy lattice model for the folding of proteins into two almost identical structures, one entangled and one not. As expected, the folding time is longer when random sequences folds into the entangled structure. This holds also under an evolutionary pressure simulated by optimizing the folding time. It turns out that optmized protein sequences in the entangled structure are in fact characterized by frustrated interactions at the closures of entangled loops. This phenomenon is much less enhanced in the control case where the entanglement is not present. Our findings, which are in agreement with experimental observations, corroborate the idea that an evolutionary pressure shapes the folding funnel to avoid topological and kinetic traps.

## 1. Introduction

The biological function of most proteins requires them to fold into a well-defined native state, implying that both structure maintenance and efficient folding are kept under selective pressure by evolutionary processes [1]. In particular, a direct experimental evidence, pointing to some degree of folding rate optimization throughout evolution, was recently provided for ribonuclease H, using ancestral sequence reconstruction [2]. Bio-informatics analyses had also uncovered similar evolutionary signals already two decades ago for several folds [3], and more recently for a large catalog of protein domains [4].

The latter study was based on the well known empirical correlation between experimentally measured folding rates of proteins and simple descriptors of the structural organization of the native state [5]. More general features of the folding mechanism are as well dictated by the overall topology of the native state [6]. In fact, contact order [7] and other related descriptors are based on the topological properties of the network formed by pairs of residues that are nearby in the three-dimensional space [8]. The simpler the network, the faster the predicted folding. The topology of the network of contacts, however, does not necessarily capture the topology of the protein backbone seen as a curve in the three-dimensional space, and the possible formation of knots and other entangled motifs.

The discovery of knots in few proteins [9] came indeed as a surprise because they seem an unnecessary complication for the folding. Their presence could be related to some biological function or stability requirement [10,11], and the mechanisms allowing the dynamics to thread the protein backbone to form knots are under intense investigation [12,13,14,15,16].

After knots, it was realized that other topological motifs may tangle the three-dimensional structure of some proteins. These include knotoids [17], slipknots [18], lassos [19,20], pokes [21] and other forms of entanglement [22,23,24,25] related to the mathematical concept of linking number [26]. It is possible to quantify such linking by means of Gauss integrals [22,27,28], from which the proposed name of Gaussian entanglement [23,25]. Also this kind of intricacy may lead to a slowing down of the folding, as suggested by the significant correlation between Gaussian entanglement and folding rates [23]. Interestingly, the Gaussian entanglement and the contact order can be combined to improve the predictions of folding rates [23].

Recently, it was discovered that entangled loops (i.e., looped portions of a protein with large Gaussian entanglement with another portion of the same protein) appear in roughly one third of known single domain proteins [25], a much larger fraction than that of knotted proteins [10]. Moreover, the amino acids at the closures of entangled loops have a mutual attraction which is on average, weaker than in the set of all closures [25]. A plausible explanation of the statistical lack of stable closures for entangled loops is that they would require a complicate threading by another part of the protein after their early formation. It is likely better for the folding dynamics to perform the closure of entangled loops as late as possible. This hypothesis is corroborated by an asymmetry in the position of entangled loops with respect to the chain portion they are entangled with, the thread, such that the latter is found more frequently on the loop N-terminal side [25]. In the context of cotranslational folding [29], this would imply that entangled loops are synthesized at the ribosome, and hence folded, on average later than the thread.

In this work we aim at understanding if the weak closure of entangled loops can be interpreted, at least in principle, as the result of a selective pressure that optimizes the folding rate. We do this within a simple toy model where short protein chains are defined on a face-centered cubic (fcc) lattice, forming a population with random initial amino acid sequences that are then subject to an evolutionary process. We consider two different putative native states sharing exactly the same ground state energy (for similar sequences) and similar network topologies. However, one is characterized by a large Gaussian entanglement (in the form of two concatenated loops), whereas the second one presents no significant entanglement and is used as a negative control. This simple model allows a sufficiently quick repetition of the folding dynamics for many protein copies within a structure-based approach [30]. At every step of the evolutionary process, the sequence with the longest folding time is replaced by another sequence, so that the population evolves toward a state where the entangled loops are indeed on average more weakly bound at their closures than other non-entangled loops in the same structure. This effect is much less enhanced in the negative control case, when all loops in the native structure are not entangled. A similar evolutionary strategy was used to assess kinetically optimal pathways for complex lasso proteins [31].

## 2. Results

**Figure 1:**
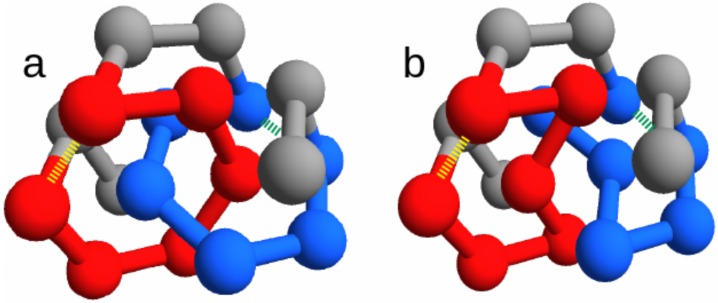
(**a**) Entangled native state. (**b**) Non-entangled native state. The putative “twin” native states are two self-avoiding walks on the fcc lattice with N=18 sites. The two loops shown in red and blue are concatenated in the entangled structure, and not concatenated in the non-entangled structure. The amino acids at the ends of each loop form the contacts (dashed yellow and green lines) whose energy is studied in this work.

In our toy model for protein chains, we consider a structure-based energy function with a sequence-dependent energy (Section 4.1 for details). Once a native structure is chosen to define the energy function, all sequences in the model will have that structure as a ground state, with a sequence-dependent ground state energy. In this study, we consider two putative native states. One state is entangled, with two concatenated loops, whereas the second “twin” state is non-entangled (Figure 1).

Despite the different overall topology, the two twin states display a very similar contact network topology (compare Table 1 and Table 2). In fact, one of the two states can be converted into the other by just switching the spatial positions of two particular amino acids, so that only a few contacts are rewired. The overall energy, however, can be kept exactly the same, upon also switching the corresponding amino acid types (Section 4.2 for details).

We focus on the two energies of the contacts involved in the closures of the loops, which can be either the concatenated loops in the entangled native state or the corresponding loops in the “twin” non-entangled state. These are represented as dashed lines in Figure 1, joining amino acid 3 with 8 and amino acid 11 with 16. Due to the symmetry of the conformations, the two contacts are in equivalent positions. Therefore, for any sequence *s*, we can distinguish the energies $V_{1}\left(s\right)>V_{2}\left(s\right)$ of the contacts with, respectively, the weaker (higher energy) and the stronger interaction (more stable due to lower energy).

### 2.1. Concatenated Loops Slow Down the Folding of Random Sequences

**Figure 2:**
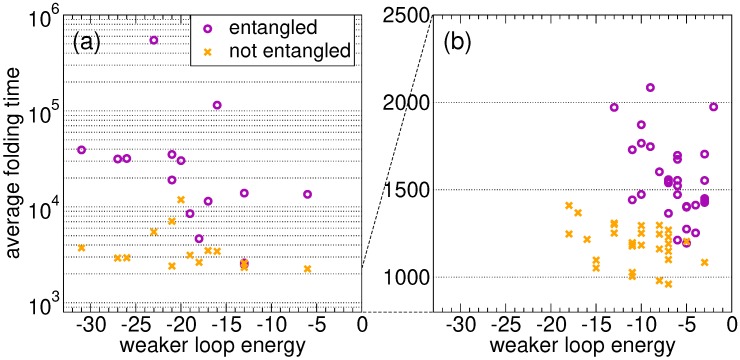
Average folding times into the entangled native state (circles) and into the twin native state without entanglement (crosses), as a function of the weaker of the contact energies involved at the ends of the two loops ($V_{1}$). (**a**) 15 independent random sequences; (**b**) fastest proteins after G=1000 generations of the evolutionary process for 31 independent replicas. Evolution leads to a dramatic drop in time scales (note the log scale for the random sequences), yet the entangled proteins, with respect to their non-entangled twins, still fold more slowly. Note also that the evolved entangled proteins have on average more unstable energetic closures of the loops.

We begin by comparing the average folding times of random sequences that fold onto the entangled native state, shown in Figure 1a, with those of their twin sequences that fold onto the twin non-entangled native state, shown in Figure 1b, with the same ground state energy. The random sequences $\left\{a_{1},\ldots ,a_{p},\ldots ,a_{q},\ldots ,a_{N}\right\}$, assigned to the entangled native state, are sampled with a uniform distribution over all 20 possible amino acid types. The related twin sequences can then be defined as $\left\{a_{1},\ldots ,a_{q},\ldots ,a_{p},\ldots ,a_{N}\right\}$. We perform n=100 runs to estimate the average folding times for each considered sequence (Section 4.3 for simulation details). This procedure is repeated for 15 random sequences and their twins. Figure 2a shows the average folding time as a function of the weaker energy $V_{1}\left(s\right)$. As expected, the proteins that fold onto the entangled state need on average much more time than their twins. For random sequences, in the absence of an evolutionary process, the folding time is not correlated with the loop closure energy in both the link and the no-link case. A similar conclusion can be drawn for the stronger energy $V_{2}\left(s\right)$. We present the results for 15 random sequences only, because the difference between the two ensembles shown in Figure 2a is already apparent (note the log scale on the folding time axis).

### 2.2. Folding Time Optimization Results in Slightly Lower Average Energies for the Concatenated Protein Structure

After having established the folding kinetic properties of random sequences, we now study the outcome of an evolutionary process that optimizes the average folding time for the resulting sequences (Section 4.4 for more details on the simulated evolutionary process).

For a given choice of the putative native state, the evolutionary process is simulated for S=31 independent replicas. In each replica, Z=100 proteins with random initial sequences are evolved for a total of G=1000 evolutionary steps, or generations. At the end of the process, the final ensemble of each replica consists of *Z* optimized sequences with statistical properties distinguished from the initial random ones.

We simulate the evolutionary process with either the entangled conformation (Figure 1a), or its non-entangled “twin” (Figure 1b), chosen as the respective native state. We focus on the properties of the “best” protein $\omega_{s}^{*}$, i.e., the protein with the lowest average folding time $\tau_{s}=\langle \tau (\omega_{s}^{*})\rangle$, within each system 1≤s≤S.

The resulting native energy per residue *E* of such proteins, averaged on the ensemble of *S* independent replicas, is slightly lower in the link case ($\langle E\rangle =-22.13\pm 0.16$) with respect to the no-link case ($\langle E\rangle =-21.76\pm 0.15$). This energy difference is significant at the level of 1.7 standard deviations (*p*-value 0.045 with a one-tailed test); it may be needed to compensate for the entropy loss caused by the rigidity due to loop concatenation [25].

### 2.3. Folding Time Optimization Promotes Weak Interactions At the End of Concatenated Loops

For all proteins with the lowest average folding time, the latter is shown in Figure 2b as a function of the energy $V_{1}\left(s\right)$ of the weakest closure (a similar pattern is found for $V_{2}\left(s\right)$), for the *S* fastest proteins evolved on the entangled native state and for the *S* fastest proteins in the no-link case. Clearly, the former ones on average fold more slowly than the latter ones. Most importantly, proteins evolved on the entangled native state are characterized by higher $V_{1}$ values, i.e., the closures of concatenated loops are less stable as a result of the evolutionary process, even if their overall native energy is lower (Section 2.2).

On average, over the *S* independently replicated evolutionary processes, we find $\bar{\tau }=\frac{1}{S}\sum_{s}\tau \left(s\right)=1560\pm 40$, ${\bar{V}}_{1}=\frac{1}{S}\sum_{s}V_{1}\left(s\right)=-6.5\pm 0.5$, ${\bar{V}}_{2}=\frac{1}{S}\sum_{s}V_{2}\left(s\right)=-11.4\pm 0.7$, in the presence of an entangled native state. In the no-link case, we get instead significantly lower values for all quantities (at a level of, respectively, 8.3, 5.1, 5.4 standard deviations in the different cases): $\bar{\tau }=1190\pm 20$, ${\bar{V}}_{1}=-10.9\pm 0.7$, ${\bar{V}}_{2}=-18.4\pm 1.1$.

**Figure 3:**
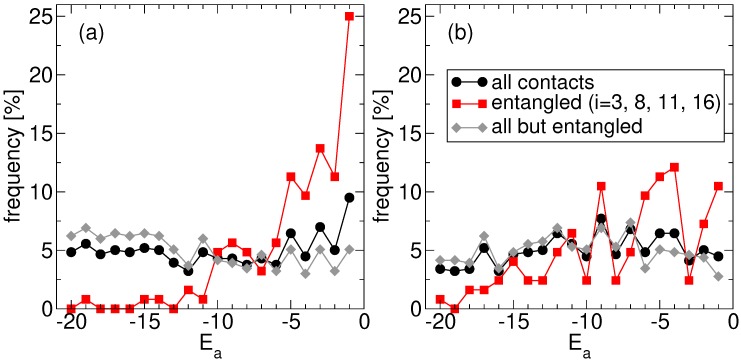
Frequency of amino acid single potentials for (**a**) protein with entangled loops and (**b**) its twin without entanglement, collecting the statistics of proteins at the end of the evolutionary process. The three curves show the frequency regardless of the position along the chain, the frequency at the 4 sites closing the loops, and the frequency in the complementary set of sites.

Consistently, our findings reveal also that folding time optimization on the entangled native structure leads to sequences where concatenated loops are closed by amino acids that are less hydrophobic than the average one. This is made apparent in Figure 3a, where we focus again on the ensemble of the *S* fastest proteins found at the end of the corresponding independent replicas of the evolutionary process. We plot the frequency observed for each amino acid type *a* (the corresponding hydrophobicities are negative integers, $-20\leq E_{a}\leq -1$ in our toy model, Section 4.1): (i) regardless of its position along the chain, (ii) at one of the 4 sites at the ends of the two concatenated loops (dashed lines in Figure 1a), and (iii) at the complementary N−4 sites. Case (i) and (iii) show that, on average, all amino acids are equally frequent, consistently with the sampling of amino acid types used in the evolutionary process. Note that, in principle, one might have expected an overall bias towards more hydrophobic residues, based on the naive expectation that the stronger the interactions the faster they form, but this is not the case. The presence of an essentially flat distribution over all residue types also suggests that our results do not depend on the specific choice of the simulation temperature nor on possibly different definitions of folding temperature [32] on which that choice is based (Section 4.3). The slight difference between (i) and (iii) is due to the inclusion in (i) of the statistics (ii) of the 4 special sites, which shows a significant departure from the flat profile. Indeed, due to the evolutionary pressure promoting fast folding, the most hydrophobic residues are selected against at the end of concatenated loops (amino acids with $E_{a}<-10$ are nearly absent), whereas hydrophilic ones are instead found much more frequently (the distribution has a large peak in correspondence of the more hydrophilic amino acid with $E_{a}=-1$).

It is important to check that this trend is actually due to the presence of two concatenated loops and not simply to the overall arrangement of the native structure. The corresponding frequencies observed for the amino acid types in the no-link case, where the 4 “twin” sites in the non-entangled state (connected by dashed lines in Figure 1b) are either singled out or excluded, are shown in Figure 3b. The residue type selection observed in the link case is much stronger than in the no-link case. Nevertheless, a similar, albeit much slighter, trend is present also in the latter case, with hydrophilic residues found more frequently than hydrophobic ones at the 4 special sites. The most hydrophobic residues can anyhow still be found in a significant amount.

## 3. Discussion

Within a toy model for short protein chains, we simulated an evolutionary process where folding time is optimized for a given native structure. Coarse-grained structure-based models are commonly used to study folding kinetics, in particular in the context of knotted and entangled proteins [24,31,33,34,35]. We focus on folding time optimization because we want to test the effect of a specific evolutionary pressure on the folding kinetics due to the presence of entangled motifs. In general, the evolutionary fitness of a protein is obviously related to its biologic functionality, of which fast and reproducible folding is only a prerequisite.

In order to better understand the role of entanglement, we considered two similar native structures that are related by a subtle rewiring of few chain bonds and thereby differ only for the presence of a pair of concatenated loops in just one of them (Figure 1). For any given sequence that folds onto the entangled native structure, a “twin” sequence can be obtained by switching two amino acid types, having the same ground state energy onto the non-entangled native structure.

Given the fundamental role played by the topology of protein structures [6], we expect that even the extremely coarsened approach used in this paper should detect the differences in the folding properties due to the presence of entangled motifs. Ultimately, the reliability of any modeling approach needs to be evaluated against its ability in reproducing observed data. As a matter of fact, our results reproduce sequence patterns related to the presence of entangled motifs that were detected by analyzing single-domain protein structures [25].

Namely, the evolutionary process leads to optimized sequences whose amino acids are enriched in hydrophilic residue types at the end of concatenated loops, when the latter are present in the native structure (Figure 3). The results obtained within the toy model thus corroborate the hypothesis that the need to perform a fast and smooth folding process has selected amino acid sequences where some degree of frustration, in the form of unfavorable amino acid pair stability, is allowed. This energetic frustration is localized at the ends of concatenated/entangled loops, allowing to overcome the topological frustration implied by the presence of entangled motifs, in keeping with the principle of minimal frustration [36,37].

The crucial role of loop concatenation is benchmarked against the results obtained for the non-entangled native structure. A residual selection of hydrophilic residues is observed also in this case, hinting that the evolution of weak interactions to allow fast folding may be a feature non restricted to the closures of entangled loops. At any rate, the observed enrichment in hydrophilic residues is markedly weaker than for the entangled structure (Figure 3). At the same time, a bias, if any, is observed instead towards lower overall native energies of the evolved sequences for the entangled native structure (Section 2.2).

In general, folding is on average much slower in the presence of concatenated loops, as expected. This holds true when comparing the ensemble of sequences evolved independently on the two native structures (Figure 2b), and also when comparing the folding of random sequences onto the entangled native structure with their “twin” sequences on the non-entangled structure (Figure 2a).

To sum up, our results support the following picture: given a specific three dimensional arrangement of residues in the native structure, if evolution selects sequences enhancing the folding rate, a crucial byproduct is the removal of strong stabilizing interactions at the ends of loops, in particular at the end of concatenated loops that presumably need to be formed in the latter stages of the folding process. Future work should obviously investigate whether this results hold for more fine-grained protein models and/or for bigger protein chains.

## 4. Materials and Methods

### 4.1. Protein Chain Model

We model a protein as a *N*-site self-avoiding walk on the fcc lattice. Each residue i∈{1,2,…,N} carries an amino acid type $a_{i}$ with hydrophobicity quantified from $E_{a}=-a$ for a∈{1,2,…,19,20}. Hydrophilic amino acids have $E_{a}$ closer to zero and thus form weaker binding with other residues, as described next.

Non consecutive residues i,j (|i−j|>1) form a contact if they are nearest neighbors on the fcc lattice. We follow a structure-based Go-like approach [30] and assign an energy to any such contact as

(1) $$V_{i,j}=E_{a_{i},a_{j}}\cdot \Delta_{i,j}=\left(E_{a_{i}}+E_{a_{j}}\right)\Delta_{i,j}.$$

Here $\Delta_{i,j}=0,1$ is the native connectivity matrix (the contact map) in the Go-like model, namely $\Delta_{i,j}=1$ only if the contact i÷j is present in the native state. The energetic contribution $E_{a_{i},a_{j}}\equiv E_{a_{i}}+E_{a_{j}}$ is the simplest linear combination of the amino acid hydrophobicities. Note that all contact energies are attractive in our model, so that any given structure with a non zero connectivity matrix is the ground state for all sequence choices. The overall energy for a given chain configuration Γ with amino acid sequence ${\left\{a_{i}\right\}}_{i=1}^{N}$ is obtained by summing over all nearest-neighbor interactions on the lattice among non consecutive residues along the chain:

(2) $$E\left(\Gamma ,\left\{a_{i}\right\}\right)=\sum_{j>i+1}V_{i,j}I_{i,j}\left(\Gamma \right)=\sum_{j>i+1}\left(E_{a_{i}}+E_{a_{j}}\right)\Delta_{i,j}I_{i,j}\left(\Gamma \right).$$

where $I_{i,j}\left(\Gamma \right)=1$ if sites $\Gamma_{i}$ and $\Gamma_{j}$ are nearest neighbors and $I_{i,j}\left(\Gamma \right)=0$ otherwise.

Note that the simple additive form Equation (1) of the pairwise interaction potential was shown to capture much of the statistical variability of the interaction parameters derived by Miyazawa and Jernigan in a knowledge-based approach [38].

### 4.2. Native Conformations

We consider two alternative native state configurations for short self-avoiding chains (N=18), shown in Figure 1. Both structures are the ground states for any sequence choice, in the corresponding structure-based models defined by Equation (2). They are non-degenerate only up to mirror images, since we do not consider terms that break the chiral symmetry. The list of their contacts in the native conformation is presented in Table 1 for the entangled protein and in Table 2 for the twin without entanglement.

The choice of short chains allows fast simulations, at the same time leaving the possibility for a non trivial topological structure that exhibit a pair of concatenated loops. We choose this entangled structure, $\Gamma_{l}$, as the main object of our study (see Figure 1a).

The fcc lattice allows to build an almost identical non-entangled structure, $\Gamma_{nl}$ (through the text we called it the “twin” structure), where no pair of loops is concatenated (Figure 1b). The twin non-entangled structure $\Gamma_{nl}$ can be obtained by just switching the spatial positions ($r_{p}↔r_{q}$) of the two residues p=6 and q=13 in the entangled structure $\Gamma_{l}$. Note that this entails a rewiring of chain connectivity, consistently with the change in the overall topology. With a simple notation, the two twin structures are labeled as “link” and “no link” in the figures.

If, in addition, one performs a similar switch ($a_{p}↔a_{q}$) between the corresponding amino acid types in the sequence, all amino acid types turn out to be kept in the same three-dimensional positions. However, the values of 4 contact energies are modified under the combined structural and sequence switches, in correspondence of the interacting pairs affected by chain rewiring.

Nevertheless, the simple form Equation (1) for the amino acid pairwise interaction potential ensures that, the sequence $\left\{a_{i}\right\}$, with ground state energy $E\left(\Gamma_{l},\left\{a_{i}\right\}\right)$ on the entangled structure, will have exactly the same ground state energy as the switched “twin” sequence $\left\{a_{i}^{\prime }\right\}$ on the non-entangled twin structure: $E\left(\Gamma_{l},\left\{a_{i}\right\}\right)=E\left(\Gamma_{nl},\left\{a_{i}^{\prime }\right\}\right)$. Thanks to the above property, the non-entangled structure does not have any energetic advantage with respect to the entangled structure $\Gamma_{l}$ during the folding of selected sequences.

Finally, we note that there is an additional symmetry for both structures, because the inversion of the chain direction (i↔N−i+1) produces its mirror image. Accordingly, the detected evolutionary signals will not depend on the asymmetry in the location of the concatenated loops along the chain.

### 4.3. Folding Simulations

**Figure 4:**
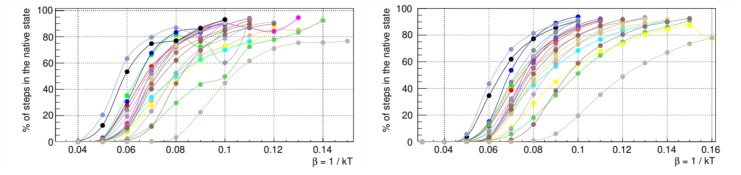
Fraction of configurations in the native states as a function of the inverse temperature $\beta =1/k_{B}T$, for proteins with entanglement (left) and without entanglement (right). Each curve is for a given random sequence. The mean folding inverse temperature is found by averaging the points where the curves cross the 50%. The inverse temperature used in the folding simulations within the evolutionary process is fixed to this value.

The time trajectory of each protein in the conformation space is initialized from a random high-temperature configuration. The temperature is switched at time t=0 to a value T=1/β=1/0.071≃14.1 (with units in $k_{B}=1$) that was determined by averaging the folding thermodynamics properties of random sequences (Figure 4). This temperature leads to the eventual folding of the protein, a stochastic process that we simulate n=100 times. Each realization 1≤α≤n for a given protein ω takes place in a time $\tau_{\alpha }\left(\omega \right)$ and an average folding time is then evaluated as $\langle \tau (\omega )\rangle =\frac{1}{n}\sum_{\alpha =1}^{n}\tau_{\alpha }\left(\omega \right)$.

**Figure 5:**
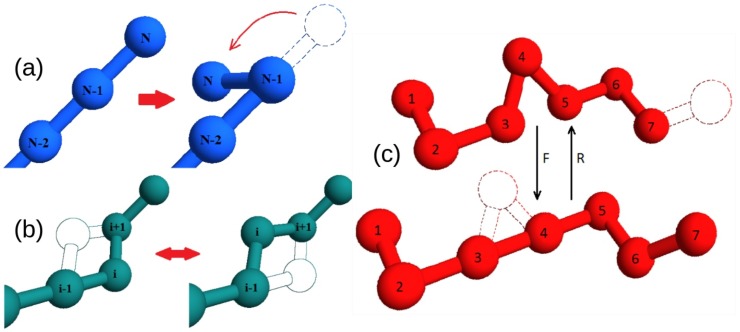
Illustration of the types of moves used in the simulations: (**a**) end-flip (frequency P=2/19, the figure illustrates the random choice at the end with site *N*), (**b**) crankshaft or internal flip (P=16/19), (**c**) reptation (P=1/19). In reptation, to satisfy detailed balance, the frequency of attempted moves in the two directions satisfy $W^{R}=3W^{F}$ (“F” and “R” following the notation in the figure) because there are 12 possible points for the added end vs only 4 internal sites for the added corner. The internal flip is in one out of the 4 possible sites if the corner is of $60^{\circ }$ or $90^{\circ }$, while only two sites are allowed when the corner is $120^{\circ }$. All attempted moves are then validated with self-avoidance constraints and eventually accepted with a Metropolis algorithm.

Protein time dynamics is simulated thanks to a set of Monte Carlo moves, including both local (crankshaft and end-flip) moves and a global sliding/reptation move, which have been carefully implemented to satisfy detailed balance (Figure 5).

Time is measured in units of Monte Carlo sweeps, one sweep containing a fixed amount M=N+1 of Monte Carlo moves. Local crankshaft and end-flip moves are selected at random with probability N/M and the global reptation move is chosen with probability 1/M to meet the intuition that a global rearrangement of the backbone is less likely to occur than a random local displacement. We include the global sliding move to endow the dynamics with the chance of threading a portion of the backbone through an already formed loop. The use of lattice Monte Carlo moves to study folding kinetics and the identification of Monte Carlo sweeps with folding time are well established in the field [35].

### 4.4. Evolutionary Process

Z=100 protein sequences, each representing an organism, are involved in the evolutionary process. All sequences are assumed to fold to a fixed native structure according to the Go-like model defined in Equation (2). The native structure can be chosen as either the entangled structure in Figure 1a, or the twin non-entangled structure in Figure 1b. At beginning of the process, protein sequences are initialized by choosing randomly each amino acid with a uniform probability across all 20 possible types. During the process, we evaluate the average folding times for all proteins as described in Section 4.3. The longest folding time is associated with the lowest fitness of the organism hosting that protein. For simplicity, this just leads to its extinction, and its position in the niche is occupied by another element. For its replacement we follow this procedure: with probability $p_{r}=1/3$ a completely new random sequence of amino acids, sampled with uniform probability as above, is assigned to the newborn; otherwise the sequence of the faster folder is copied with partial fidelity, i.e., amino acids are kept the same with probability $p_{c}=0.9$ and uniformly sampled at random otherwise. The protocol for generating the new sequence thus includes the priority gained by the organism with the best fitness (yet allowing mutations of its genome) to populate the empty slot, but also the possibility of a random entrance of brand new organisms in the empty niche.

## Figures and Tables

**Table 1:** For the entangled protein structure (Figure 1a), pairs i÷j for which $\Delta_{i,j}=1$. Underlined pairs refer to the contacts at the ends of loops discussed in this work. Pairs in bold-face (10 over 35) refer to the contacts that are not present in the non-entangled “twin” (Table 2).

Contacting Residue Pairs i÷j in the Entangled Structure						
1÷4	2÷9	4÷13	6÷13	6÷18	7÷17	10÷17
1÷5	2÷12	5÷13	6÷14	7÷9	7÷18	11÷16
1÷12	2÷13	5÷14	6÷15	7÷10	8÷13	11÷17
1÷13	3÷8	6÷11	6÷16	7÷12	9÷12	14÷18
2÷8	3÷13	6÷12	6÷17	7÷13	10÷12	15÷18

**Table 2:** For the twin protein structure (Figure 1b) without link, pairs i÷j for which $\Delta_{i,j}=1$. Underlined pairs refer to the contacts at the ends of loops discussed in this work. Pairs in bold-face (10 over 35) refer to the contacts that are not present in the entangled structure (Table 1).

Contacting Residue Pairs i÷j in the Non-Entangled Structure						
1÷4	2÷8	4÷6	6÷13	7÷13	10÷17	13÷16
1÷5	2÷9	5÷13	6÷14	7÷17	11÷13	13÷17
1÷6	2÷12	5÷14	7÷9	7÷18	11÷16	13÷18
1÷12	3÷6	6÷8	7÷10	9÷12	11÷17	14÷18
2÷6	3÷8	6÷12	7÷12	10÷12	13÷15	15÷18

## Abbreviations

The following abbreviations are used in this manuscript:

fcc	face-centered cubic
